# Analyzing the Centers for Disease Control and Prevention Mortality Data Using Weekly Exceedance in Mortality Count and Weekly Change in Mortality Indicator: A Time Series Study

**DOI:** 10.1002/hsr2.71235

**Published:** 2025-09-14

**Authors:** Aditya Chakrabarty, Mohan D. Pant

**Affiliations:** ^1^ Department of Epidemiology, Biostatistics, & Environmental Health, Joint School of Public Health Old Dominion University Norfolk Virginia USA

**Keywords:** cause of death (COD) data matrix, CDC provisional death counts, multivariate time‐series forecasting modeling, weekly change in mortality indicator (*WCMI*), weekly exceedance in mortality count (*WEMC*)

## Abstract

**Background and Aims:**

Cause‐specific mortality (CSM) count prediction plays a vital role in the context of public health policy. In this study, we introduce a new analytical approach, which is divided into three phases to answer specific questions regarding CSM due to 14 specific causes by computing different simple, compound, and conditional probabilities.

**Methods:**

A multivariate time series forecasting model was developed using the CDC weekly mortality count data. A binary data matrix was constructed for 14 causes of death (COD) as a function of weeks by combining the observed and forecasted mortalities. We introduced two new concepts: Weekly Exceedance in Mortality Count (*WEMC*) and Weekly Change in Mortality Indicator (*WCMI*), which were instrumental in computing various probabilities relating to all the CODs. To test the null hypothesis of no association between the COD and *WEMC* a chi‐square test of independence was conducted whereas Cramer's V statistic was used to check the strength of the association. Wilcoxon rank sum test, and correlation indices were used to validate the method.

**Results:**

The results of chi‐square test of independence indicated that there was no statistically significant association between COD and *WEMC* (*p* = 0.79). Furthermore, the effect size of this association between COD and *WEMC* was very small (Cramer's V = 0.055). The results of Wilcoxon rank sum test indicated that there was no statistically significant difference between the observed and forecasted counts (*p* = 0.11) confirming the consistency of our analytical method. Probabilities associated with *WCMIs* were also computed as an illustration of the analytical method.

**Conclusion:**

Utilizing this analytical approach, researchers and policymakers can compute the probabilities of any number of desired events related to different COD which can be helpful for public health interventions, resource allocation, informed decision‐making and risk assessment, by controlling the underlying attributes responsible for the probabilities to surge and plummet.

**Abbreviations**
COD: cause of death
CSM: cause‐specific mortality
MTM: multivariate time‐series model
RV: random variable
*WCMI*
: weekly change of mortality indicator
*WEMC*
: weekly exceedance in mortality count

## 1 Introduction

Analyzing mortality counts is crucial for understanding patterns in disease prevalence and informing public health interventions [1]. Researchers can utilize mortality count data from the U.S. Centers for Disease Control and Prevention (CDC) (https://www.cdc.gov/) to conduct probabilistic analyses and gain a deeper understanding of disease prevalence trends. This technique of probabilistic analyses enables a comprehensive assessment of the effects of diseases such as COVID‐19 on public health. This approach can also provide a valuable tool for evaluating the effectiveness of public health interventions and identifying areas where additional resources or targeted interventions may be needed. By incorporating mortality count data from the CDC into their analysis, researchers can develop a more accurate understanding of disease prevalence patterns and make informed decisions regarding public health interventions and resource allocation [2, 3, 4].

The concept of excess mortality has been a subject of considerable scholarly interest in recent years, especially in the aftermath of the COVID‐19 pandemic. Excess mortality refers to the disparity between the observed and expected number of deaths in a population, based on historical data. This topic has been extensively researched, with substantial literature investigating its underlying causes, its implications, and the approaches employed to address its impact on public health policy and decision‐making [5]. Researchers have examined the influence of demographic, socioeconomic, and healthcare‐related factors on excess mortality patterns [6]. For example, studies have documented the substantial racial disparities in excess mortality experienced during the COVID‐19 pandemic, with certain minority populations bearing disproportionately higher rates of excess deaths [7]. Molbak et al. [8] conducted a country‐specific analysis of weekly excess mortality among older adults, identifying a significant upward trend in all‐cause excess mortality, primarily among the elderly, in 13 of the 16 European countries studied. In a comparable analysis of excess mortality among the elderly across 12 European countries, the authors found that the impact of influenza in Europe differs from that observed during the recent pandemic and post‐pandemic periods. The resurgence of influenza A (H3N2), compounded with the impact of a cold snap, may contribute to the observed excess mortality among the elderly [9]. The COVID‐19 pandemic significantly affected global public health, leading to unprecedented excess mortality across various regions [10]. Beyond the direct mortality attributed to COVID‐19, the pandemic caused disruptions in healthcare delivery systems, contributing to increased mortality rates across a range of other non‐COVID‐19 medical conditions [11, 12]. In the context of many chronic diseases, including COVID‐19, researchers used excess mortality as a benchmark for measuring the impact of the disease. According to Beaney et al. [13], excess mortality, along with cause‐specific mortality can be useful to measure the trends within and between countries and should be considered as a measure that encompasses all causes of death (COD) and provides a metric for the overall mortality impact of COVID‐19.

Leveraging data‐driven global mortality surveillance, exceedance probability estimation provides a scalable approach to assessing health risks across diverse populations and healthcare infrastructures. By identifying notable changes in mortality trends, this probability estimation can enable prompt public health measures. The significance, supported by credible statistics, is broken down by certain COD, below.

Each year, approximately 805,000 people in the United States experience a heart attack. Among them, around 605,000 are the people who experience their first heart attack, while about 200,000 people have prior history of heart attack. (https://www.cdc.gov/heart-disease/data-research/facts-stats/index.html). From 106·8 (per 100,000 persons) in 2019 to 144·1 in 2020 and 148·3 in 2021, there was a notable increase of almost 30% in mortality among the 4·25 million diabetic mellitus related fatalities that occurred between 2006 and 2021 [14]. In 2021, chronic lower respiratory conditions, such as COPD, accounted for over 147,000 fatalities in the United States, making them the sixth most common cause of death, according to the CDC (https://www.cdc.gov/nchs/fastats/copd.htm). The overall burden of influenza (flu) for the 2023–2024 flu season was an estimated 40 million flu‐related illnesses, 18 million flu‐related medical visits, 470,000 flu‐related hospitalizations, and 28,000 flu‐related deaths (https://www.cdc.gov/flu-burden/php/data-vis/2023-2024.html). According to the World Health Organization (WHO), 14.9 million excess deaths were associated with the COVID‐19 pandemic in 2020 and 2021 (https://www.who.int/news/item/05-05-2022-14.9-million-excess-deaths-were-associated-with-the-covid-19-pandemic-in-2020-and-2021).

Extreme flu seasons commonly overwhelm hospitals and intensive care units when mortality rates surge past critical levels. Anticipating these dangerous peaks allows healthcare providers to respond proactively and distribute resources judiciously. While conventional time series models excel at capturing long‐term mortality trends, they may lack the sensitivity to effectively identify short‐term spikes in mortality. The application of exceedance probability analysis offers a scalable approach to the early detection of substantial deviations from expected mortality patterns, thereby facilitating timely public health interventions.

Cause‐specific mortality prediction (CSMP) continues to remain an essential component of epidemiological studies. It is not restricted to geographical borders and has an equal impact on the inhabitants of a specific country/region. Analytical forecasting models play a vital role in comprehending future scenarios by taking historical data into account. Researchers have been using these analytical models to answer specific questions related to cause‐specific mortality. do Nascimento et al. [15] applied light gradient boosted machine (LGBM), and extreme gradient boosted (XGB) algorithms to predict mortality by diseases of the respiratory system (DRS) within 5 years, using socioeconomic, demographic, and health features. The study was conducted with a representative sample of older residents from São Paulo, Brazil, and the machine learning (ML) algorithms produced a high prediction accuracy. In a study by Foreman et al. [16], the authors provided an efficient, adaptable forecasting platform from which reference forecasts and alternative health scenarios can be explored in relation to a wide range of independent drivers of health, and used an autoregressive integrated moving average (ARIMA) model to forecast the life expectancy, years of life lost, and all‐cause and cause‐specific mortality for 250 COD. Shen et al. [17] used data from the National Cancer Institute's Surveillance, Epidemiology, and End Results (SEER) program (https://seer.cancer.gov/), also used in [18] and [19] in different contexts, to develop a CSMP model for patients with basaloid squamous cell carcinomas of the head and neck. According to Shen et al. [17], the prediction model's performance was good, and the model was purported to serve as a predictive prognostic tool to assist patients and physicians in clinical counseling. Yan et al. [20] developed an XGBoost machine learning model, trained on COVID‐19 data from Wuhan, China, demonstrating the capacity to predict patient mortality rates with over 90% accuracy more than 10 days in advance. Moreira et al. [21] developed a logistic regression model to predict mortality for extremely low gestational age neonates. The model's performance was assessed via the area under the receiver operating characteristic curve (AUC) and compared to validated mortality prediction models and an external cohort of neonates.

Although there are plenty of studies in the literature that use predictive or forecasting models such as ([22, 23, 24, 25, 26, 27, 28]) to predict or forecast mortality due to a specific COD, we could not find any analytical method that can be used for modeling the Weekly Exceedance in Mortality Count (WEMC) (definition 2.1) via probability distribution. Because cause‐specific mortality is a function of time, it is crucial to identify instances where mortality in the current week exceeds mortality in the past week and to calculate the probabilities for each COD.

Existing research has uncovered valuable insights into overall mortality patterns, but a critical gap remains in leveraging real‐time, mortality data to promptly identify sudden exceedances. Conventional analyses primarily focus on annual death rates, which can conceal important fluctuations that signal emerging public health threats, such as influenza outbreaks or abrupt surges in COVID‐19 cases. The proposed method of computing the probability of weekly mortality exceedances is pivotal, as it can enable the early detection of mortality spikes that could be unnoticed in more aggregated analyses. By applying this method to leading COD, we aim to enhance public health surveillance and response systems, offering a more agile and responsive approach to managing mortality risk, particularly during seasonal or pandemic‐related surges.

## 2 Methods

The study data set was extracted from the U.S. Centers for Disease Control and Prevention (CDC) https://data.cdc.gov/NCHS/Weekly-Provisional-Counts-of-Deaths-by-State-and-S/muzy-jte6. The data set lists the mortality counts (number of deaths in the U.S. due to a specific cause) across various causes from 1/4/2020 − 9/16/2023 (194 weeks). The COD considered in this study are listed below:

- DIAMEL ($C_{1}$): Mortality due to diabetes mellitus
- MALNEO ($C_{2}$): Mortality due to malignant neoplasm
- ALZ ($C_{3}$): Mortality due to Alzheimer's Disease
- INFPNE ($C_{4}$): Mortality due to influenza & pneumonia
- CLRD ($C_{5}$): Mortality due to chronic lower respiratory disease
- HD ($C_{6}$): Mortality due to disease of the heart
- CD ($C_{7}$): Mortality due to cerebrovascular Diseases
- COVIDUC ($C_{8}$): Mortality due to COVID‐19 as the underlying cause
- SEPTICEMIA ($C_{9}$): Mortality due to Septicemia
- ODRS ($C_{10}$): Mortality due to other diseases of the respiratory system
- NNSN ($C_{11}$): Mortality due to nephritis, nephrotic syndrome, and nephrosis
- SSACL ($C_{12}$): Symptoms, signs and abnormal clinical and laboratory findings mortality
- NATCAUSE ($C_{13}$): Mortality due to natural cause
- ALLCAUSE ($C_{14}$): All‐cause mortality

A multivariate time‐series model (MTM) was developed considering each of the 14 attributes (cause‐specific deaths) by taking the correlated structure into account. The MTM analysis was conducted using the prophet package of R version 4.2.1, which was released by Meta's Core Data Science team ([29, 30]). In our data set, most of the CODs show a weekly, and yearly seasonal trend. In Meta's Prophet package, weekly and yearly seasonality is usually modeled as a combination of periodic components by adjusting the parameters in the model. While developing the MTM, the weekly and yearly seasonalities were adjusted using weekly. seasonality = TRUE and yearly. seasonality = TRUE so that the prophet package can automatically incorporate the weekly and yearly seasonal patterns in the model. Each cause represents a stochastic realization of time with a significant correlation across the variable. Hence, we conducted a multivariate time series analysis ([31]) considering each of these 14 causes.

Time series analysis is a crucial technique for examining the patterns and trends in mortality counts over time. It enables researchers to investigate changes in mortality rates and identify potential correlations with specific events or interventions. By integrating this method with the analytical approach for calculating probabilities using the CDC mortality data, researchers can develop a thorough understanding of disease prevalence and its public health implications. The MTM predicts the weekly mortality for the next 104 weeks (2 years) from 09/23/2023. Next, the forecasted values (104 weeks) were combined with the observed values (194 weeks) consisting of a total of 194 + 104 = 298 weeks of information in the combined data set. After the combined data set was created, the next goal was to figure out an analytical way to address the number of instances where the mortality counts in the current week, exceeded the count in the previous week. In achieving the goal, a new indicator variable $C_{i}$ was defined in the following way:

(1) $$C_{i}=\left\{\begin{array}{l} 1,X_{\mathrm{ijt}}>X_{\mathrm{ij}\left(t-1\right)}\\ 0,X_{\mathrm{ijt}}\leq X_{\mathrm{ij}\left(t-1\right)} \end{array}\right.$$

where $X_{\mathrm{ijt}}$ is the $j^{\mathrm{th}}$ observation (mortality count) by cause *i* (*i* = 1, 2,*…*, 14) at week *t* (*t* = 1, 2,*…*, 194). That is, in our combined data set, we put $C_{i}$ = 1 for a specific cause of death (COD) $C_{i}$ (i = 1, 2,…, 14) if the mortality count in the current week *t* is greater than the mortality count of the previous week (*t* − 1). The combined data set was restructured in the following way (Table 1), where *C*_*i*_ represents the *i* ^*th*^ column of the COD data matrix. After the structural form of the COD matrix was expressed as a function of *t*, and $C_{i}$, two new terms: Weekly Exceedance in Mortality Count (*WEMC* or Ψ), and Weekly Change of Mortality Indicator (*WCMI or* $\alpha_{i}$) were defined to address the Weekly Exceedance in Mortality due to specific COD.

**Table 1:** Construction data matrix of the COD.

Week	$C_{1}$	$C_{2}$	$C_{3}$	$C_{4}$	$C_{5}$	$C_{6}$	$C_{7}$	$C_{8}$	$C_{9}$	$C_{10}$	$C_{11}$	$C_{12}$	$C_{13}$	$C_{14}$
1	1	1	1	1	1	0	1	0	0	1	0	1	1	1
2	0	0	0	0	0	0	0	1	0	0	1	1	0	1
3	0	0	0	1	1	1	0	0	0	1	1	0	1	1
·	·	·	·	·	·	·	·	·	·	·	·	·	·	·
·	·	·	·	·	·	·	·	·	·	·	·	·	·	·
·	·	·	·	·	·	·	·	·	·	·	·	·	·	·
·	·	·	·	·	·	·	·	·	·	·	·	·	·	·
195	1	1	1	1	1	1	1	1	1	1	1	1	1	1
·	·	·	·	·	·	·	·	·	·	·	·	·	·	·
·	·	·	·	·	·	·	·	·	·	·	·	·	·	·
·	·	·	·	·	·	·	·	·	·	·	·	·	·	·
·	·	·	·	·	·	·	·	·	·	·	·	·	·	·
297	0	0	0	0	0	0	1	0	0	0	0	0	0	0

Definition 2.1 Weekly Exceedance in Mortality Count (*WEMC* or Ψ): The event where the number of deaths in the current week t is greater than the number of deaths in the previous week *t* − 1, *t* = 1, 2,…, 194.

In reference to the above COD matrix, the Weekly Change of Mortality Indicator (*WCMI*) or $\alpha_{i}$ was introduced as follows:

Definition 2.2 Weekly Change of Mortality Indicator (*WCMI* or $\alpha_{i}$): The number of instances where *WEMC* occurs for cause of death (COD) *i* (*i* = 1, 2,…, 14).

From the definition of $\alpha_{i}$, it is reasonable to assume that they are independently distributed. However, a two‐tailed chi‐square test of independence with the level of significance 0.05 was performed to validate the claim of independence. The following hypotheses were constructed for testing purposes.

*H*_0_: COD and *WEMC* are independent.

*H*_1_: COD and *WEMC* are not independent.

The test of independence produced a nonsignificant *p‐*value (*p* = 0.79) implying that there is insufficient data evidence to reject the claim of independence. The result is also supported by a lower value of Cramer's V statistic (0.055), which also implies that there is no association between the COD and the *WEMC*. Hence, we statistically validated the plausibility of the independence of $\alpha_{i}s$.

From Table 1, we see that $\alpha_{i}$ ∼ *bin*(*n* = 297, ${\hat{p}}_{i}$), where

$${\hat{p}}_{i}=\frac{number\;of\;1s\;in\;the\;i^{\mathrm{th}}\mathrm{row}}{n}.$$

The 298^th^ week was discarded, as the indicators for the last week were all zero by the definition of $C_{i}$. Table 2 gives the estimated probabilities ${\hat{p}}_{i}s$ for each $C_{i}$ (*i* = 1, 2,*…*, 14); ${\hat{p}}_{i}$ denotes the probability that the number of deaths at week *t* exceeds the number of deaths at week (*t* − 1) for COD *i*. (*t* = 1, 2,*…*, 297). In other words, ${\hat{p}}_{i}$ is the probability of occurrence of *WEMC or* Ψ.

**Table 2:** Estimated probabilities corresponding to each COD.

${\hat{p}}_{1}$	${\hat{p}}_{2}$	${\hat{p}}_{3}$	${\hat{p}}_{4}$	${\hat{p}}_{5}$	${\hat{p}}_{6}$	${\hat{p}}_{7}$	${\hat{p}}_{8}$	${\hat{p}}_{9}$	${\hat{p}}_{10}$	${\hat{p}}_{11}$	${\hat{p}}_{12}$	${\hat{p}}_{13}$	${\hat{p}}_{14}$
0.475	0.478	0.498	0.478	0.431	0.431	0.498	0.468	0.451	0.465	0.464	0.683	0.482	0.467

Although $\alpha_{i}s$ are independent of each other, it is important to note that they aren't identically distributed, as they have different estimates of ${\hat{p}}_{i}s$ (*i* = 1, 2,*…*, 14). However, we can express the joint probability of $\alpha_{i}s$ in the following way:

(2) P(α1=x1,α2=x2,…,α14=x14)=∏i=114[P(αi=xi)]=∏i=11414xkpixk(1−pi)14−xk

### 2.1 Computing Probabilities Related to Sums of Independent Non‐Identical *WMCIs*

In statistics and probability theory, the idea of independent non‐identical binomial sum probability involves the calculation of probabilities linked to the combined outcomes of several independent discrete random variables following a binomial distribution. This concept is particularly useful when trying to analyze the combined outcomes of multiple events that may have different probabilities of success. By knowing the probability distributions of individual *WCMIs* or $\alpha_{i}s$, it is possible to compute probabilities of any finite combination of sums associated with $\alpha_{i}s$. Since $\alpha_{i}s$ are non‐identical, unlike the identically and independently distributed binomial random variables, a special technique is needed to find the probability distribution of any finite number of sums associated with $\alpha_{i}s$. In this section, we compute probabilities as a sum of independent non‐identical binomial random variables ($\alpha_{i}s$) for different choices of p. Analytical solutions for the probability density and distribution of non‐ identical $\alpha_{i}s$ are usually complex to find and strenuous to compute. Liu and Quertermous [32] proposed a method to find the probability distribution of the sum of independent non‐identical $\alpha_{i}s$ using the saddlepoint approximation [33]. In the present data, we have 14 CODs, and hence 14 $\alpha_{i}s$. In general, let *T* be a random variable formed by summing *N* independent non‐identical *WCMI*s or $\alpha_{i}s$, i=1,2,...,N. Then

(3) $$T_{N}=\sum_{i=1}^{N}\alpha_{i}$$

We are interested in the probability distribution of

(4) $$P\left(T_{N}=t\right)=\alpha_{1}+\alpha_{2}+\cdots +\alpha_{N}=t$$

When N=2, the above probability statement simplifies to

(5) $$P\left(T_{2}=t\right)=P(\alpha_{1}+\alpha_{2}=t)=\sum_{i=1}^{t}P\left(\alpha_{1}=i\right)P\left(\alpha_{2}=t-i\right)$$

### 2.2 Ethical Statement

The data included in this study is publicly available and does not require additional ethical approval from author's Institutional Review Board.

## 3 Results

**Figure 1:**
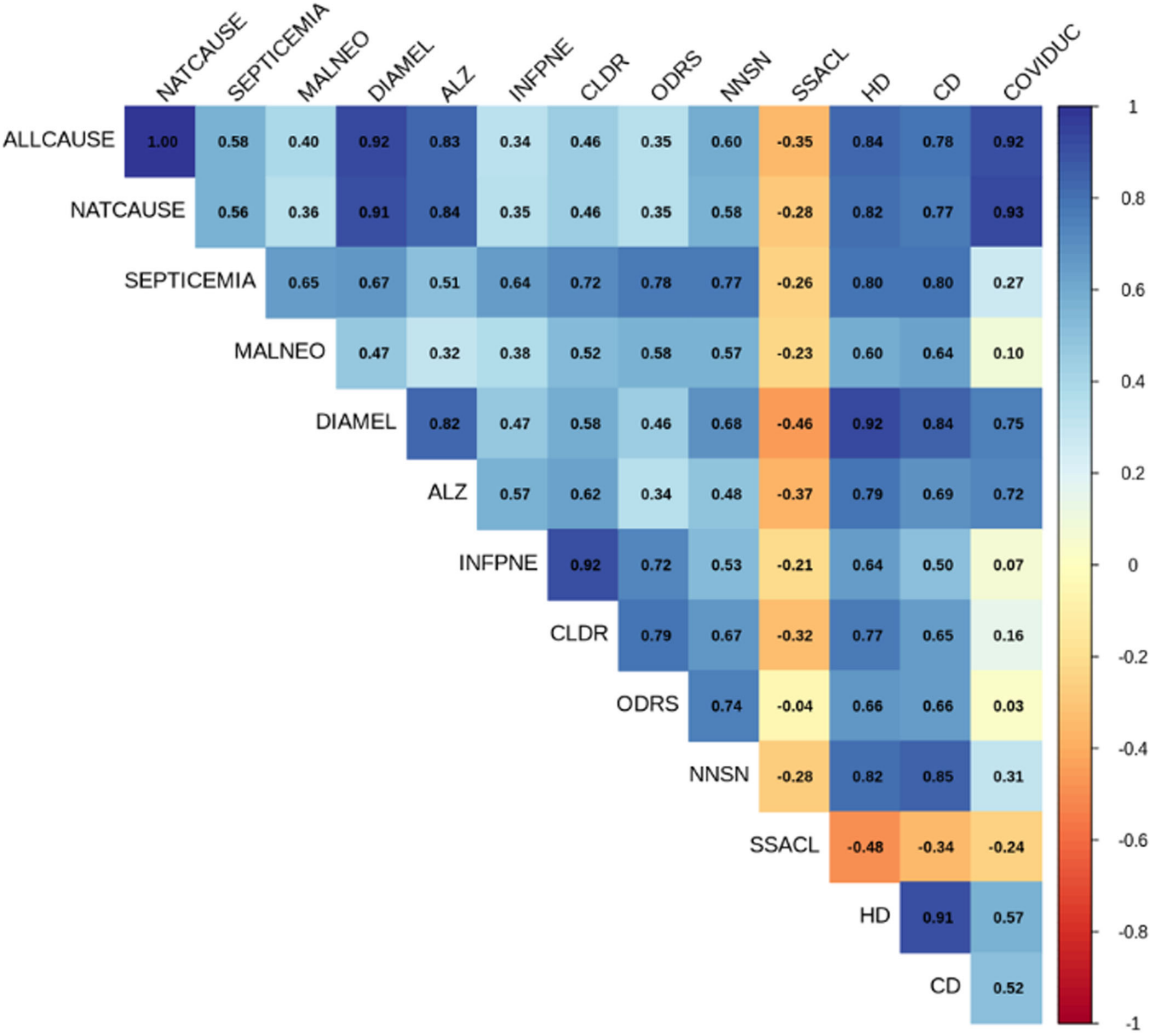
Correlation plot of mortality counts across all 14 CODs.

While some of the weekly mortalities due to CODs are highly correlated, it is important to note that $\alpha_{i}s$ are independent, and non‐identical by the data design. Figure 1 illustrates the correlation matrix of the mortality counts due to the 14 CODs.

Many such examples can be found in the literature where there is a significant association between any two ailments. The association between chronic lower respiratory disease (CLDR) with influenza and pneumonia (INFPNE) is well established [34, 35] and produced the highest correlation coefficient (0.94). Stampfer [36] discussed how cardiovascular disease (CVD) increases the risk of cognitive decline and Alzheimer's disease (AD) considering consistent epidemiological data. The correlation between diabetes and Alzheimer's disease has been a topic of interest for researchers as both conditions have shown overlapping risk factors and potential shared mechanisms [37]. From the correlation matrix (Figure 1), we see that the correlation coefficient between ALZ, and DIAMEL is approximately 0.8 attesting to the claim.

The random variable $\alpha_{i}$ has important usefulness, as it can be instrumental to estimate the prob‐ ability of any number of events/instances by using the ${\hat{p}}_{i}s$, relating to COD *i*. For example, we can answer the question: “What is the probability that the number of times Ψ or *WEMC* occurs by diabetes mellitus ($C_{1}$) is 141?” That is, can we estimate $P\left(\alpha_{1}=141\right)?$ From Table 2, we have $\alpha_{1}$ ∼ *bin* (297, 0.475). So, we can estimate different probabilities like $P\left(\alpha_{1}=141\right),P\left(\alpha_{1}\leq 141\right),P\left(\alpha_{1}\geq 141\right)$, etc. Table 3 lists some probabilities involving different $\alpha_{i}\text{s}$.

**Table 3:** Estimated probabilities of single events.

Probability statement	Probability	Probability statement	Probability
$P(\alpha_{1}\;=141)$	0.0463	$P(\alpha_{1}\geq 141)$	0.5262
$P(\alpha_{2}<136)$	0.2264	$P(47\leq \alpha_{2}\leq 94)$	0.0048
$P(\alpha_{12}>203)$	0.4717	$P(\alpha_{12}=203)$	0.0497
$P(\alpha_{9}\geq 134)$	0.5200	$P(129\leq \alpha_{9}\leq 139)$	0.4786
$P(\alpha_{7}\leq 148)$	0.5275	$P(138\leq \alpha_{7}\leq 158)$	0.7770
$P(\alpha_{8}\leq 150)$	0.9094	$P(140\leq \alpha_{8}\leq 160)$	0.4699

From Equation 2, the joint probabilities can be computed involving any number of *WCMIs* or $\alpha_{i}$. For example, if a researcher wants to know what is the chance of the joint occurrence of the event $\left(\alpha_{3}\;=\;10,\alpha_{5}=100,\alpha_{10}=70\right)$ over the period of January 4, 2020 to September 16, 2023, they can estimate the quantity, and it can be computed for any number of combinations up to 14^14^ (since 14 CODs were considered). Table 4 illustrates the estimated probabilities of some of the joint events.

**Table 4:** Estimated probabilities of joint events.

Probability statement	Probability
$P(\alpha_{1}=150,\alpha_{3}=140,\alpha_{7}=90)$	0.03 × 0.03 × 0.05 ≈ 0
$P(\alpha_{6}>130,\alpha_{7}>130)$	0.38 × 0.98 = 0.3724
$P(\alpha_{4}\geq 140,\alpha_{5}\geq 140,\alpha_{8}\geq 140)$	0.61 × 0.09 × 0.48 = 0.0263
$P(\alpha_{6}\geq 130,\alpha_{7}\geq 150,\alpha_{10}\leq 200)$	0.43 × 0.43 × 1 = 0.1850
$P(\alpha_{4}\geq 145,120\leq \alpha_{5}\geq 150)$	0.39 × 0.8362 = 0.3261

**Figure 2:**
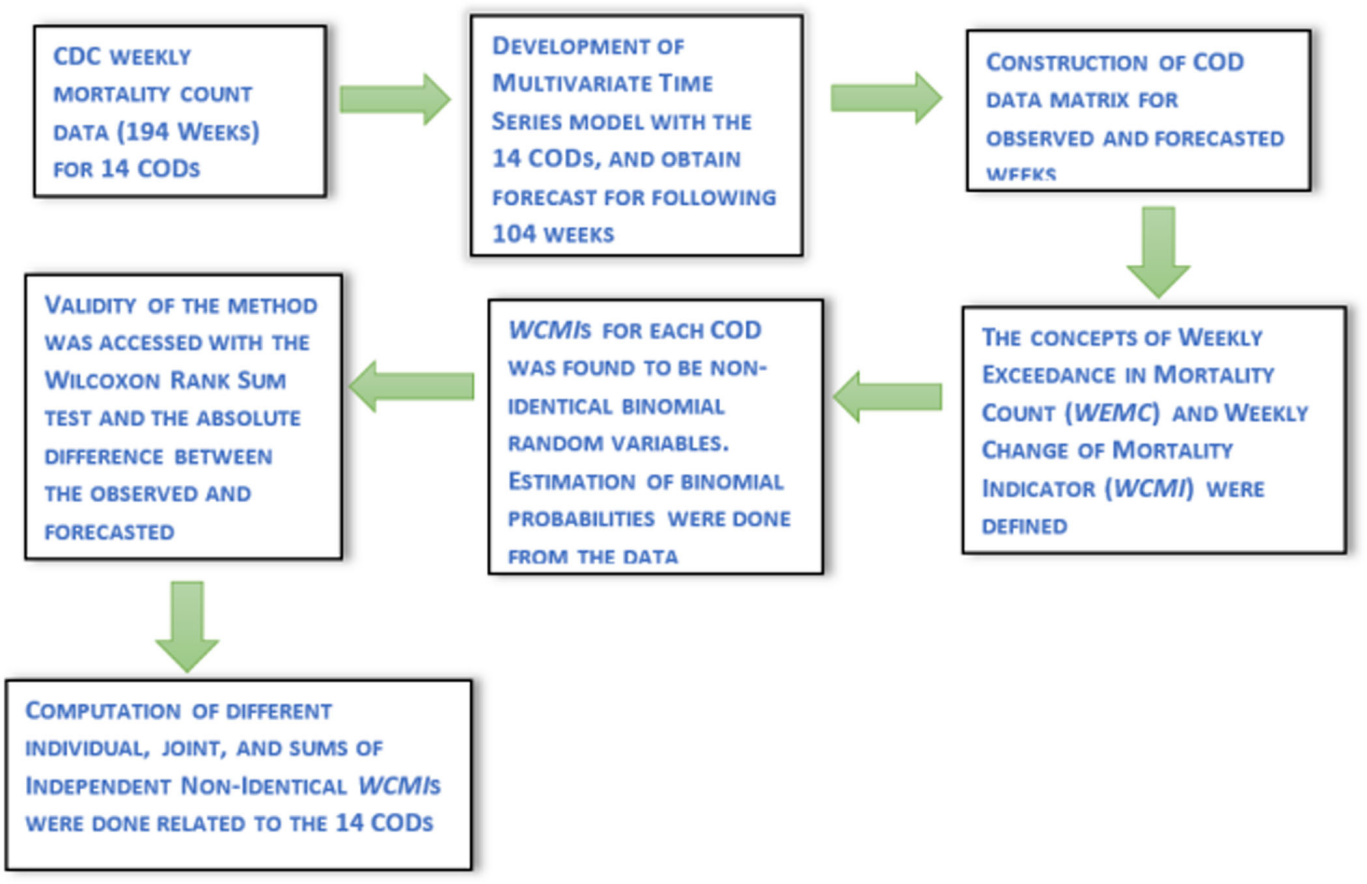
Schematic diagram of the analytical process.

However, we can estimate the joint probabilities of different *WCMIs* from Equation 2, it is true that some of the fourteen CODs are correlated, and it will be interesting to estimate the conditional *WCMIs* by accounting for the prior knowledge relating to a specific COD; that is, computing $P\left(\alpha_{i}\;=x\right|\alpha_{j}=y)$ for i≠j= 1, 2,*…*, 14. For example, from Figure 2 we see that INFPNE ($C_{4}$), and CLDR ($C_{5}$) are highly correlated (0.92). It will be interesting to compute the conditional probability of $\alpha_{4}\;=x|\alpha_{5}=y$, or vice versa, for specific values of *x* and *y*. However, due to the assumption of independence, the conditional probabilities related to any two CODs will be equal to the individual probabilities.

The computation of specific probabilities in following four tables (Tables 5, 6, 7, 8) are based on Equation (4) from Section 2.1.

**Table 5:** Computing the probabilities and cumulative probabilities of *T*_5_ for different choices of *t* when n=50, and p=0.5.

*t*	$P(T_{5}\;=t)$	$P(T_{5}\leq t)$
100	0.0003	0.0009
105	0.0020	0.0067
110	0.0083	0.0332
115	0.0227	0.1147
120	0.0413	0.2846

**Table 6:** Computing the probabilities and cumulative probabilities of $T_{5}$ for different choices of *t* when n=50, and p={0.6,0.7}.

*t*	$P(T_{5}=t),p=0.6$	$P(T_{5}\leq t),p=0.6$	$P(T_{5}=t),p=0.7$	$P(T_{5}\leq t),p=0.7$
100	8.07 × 10 − 11	1.43 × 10 − 10	0.00	7.2 × 10 − 23
105	3.75 × 10 − 9	7.12 × 10 − 9	0.00	3.1 × 10 − 20
110	1.2 × 10 − 7	2.37 × 10 − 7	0.00	8.6 × 10 − 18
115	2.40 × 10 − 6	5.35 × 10 − 6	2.55 × 10 − 15	1.6 × 10 − 15
120	3.31 × 10 − 5	8.21 × 10 − 5	3.34 × 10 − 13	2.1 × 10 − 13

**Table 7:** Computing the probabilities and cumulative probabilities of $T_{5}$ for different choices of *t* when n=50, and p={0.2,0.3}.

*t*	$P(T_{5}=t),p=0.6$	$P(T_{5}\leq t),p=0.2$	$P(T_{5}=t),p=0.3$	$P(T_{5}\leq t),p=0.3$
100	2.2 × 10 − 13	1.00	1.8 × 10 − 4	1.00
105	1.3 × 10 − 15	1.00	1.6 × 10 − 5	1.00
110	5.3 × 10 − 18	1.00	9.5 × 10 − 7	1.00
115	1.4 × 10 − 20	1.00	3.7 × 10 − 8	1.00
120	2.5 × 10 − 23	1.00	9.8 × 10 − 10	1.00

Table 5 illustrates that at a specific sample size with the increase in the values of *t* (the total number of instances assumed by the random variable $\alpha_{i}s$), at p=0.5, the joint probabilities increase. Different combinations of *α*_*i*_s can be considered to achieve a specific value of *t*. Table 6 and Table 7 illustrate the probability of the sum of five random variables (*α*_1_, *α*_2_, *α*_3_, *α*_4_, *α*_5_) for different choices of p. An increasing trend in probabilities was observed for p >0.5 (Table 6) and a decreasing trend in probabilities was observed for p <0.5 (Table 7).

The following Table 8 illustrates individual and cumulative probabilities of *T*_5_ for different combinations of n, p, and t.

**Table 8:** Computing the probabilities and cumulative probabilities of *T*_5_ for different choices of *n*, *p*, and *t*.

*n*	*p*	*t*	$P(T_{5}\;=t)$	$P(T_{5}\leq t)$
18	0.484	105	0.028	0.896
27	0.420	120	0.000	0.999
33	0.457	110	0.009	0.976
37	0.468	100	0.050	0.709
84	0.526	115	0.002	0.996

In the tables above, the probabilities were computed for illustration purposes. Different combinations of sample sizes (n), probabilities (p), and the number of events of interest (t) were considered to construct the tables. It is a way of showing the user how the computation can be implemented for any sums of independent and non‐identical *WCMIs* corresponding to any number of CODs.

### 3.1 Validation of the Proposed Technique

To validate the proposed analytical technique, a compression has been made between the observed and forecasted data. First, we computed the probability ${\hat{p}}_{\mathrm{observed}}$s for all 14 CODs based on the data from January 4, 2020 to September 16, 2023 (observed data), and then the forecasted probabilities ${\hat{p}}_{\mathrm{forecasted}}$s were computed for the 104 weeks (September 23, 2023 to September 13, 2025) of forecasted data utilizing the MTM.

In Table 9, $|{\hat{p}}_{\mathrm{observed}}-\;{\hat{p}}_{\mathrm{forecasted}}|$ measures the estimated probability differences between the observed and combined column. It is interesting to notice that the differences are diminutive, and most of these differences is less than 0.05, the stipulated level of significance.

**Table 9:** Comparing the observed and forecasted probabilities.

${\hat{p}}_{i}$	${\hat{p}}_{\mathrm{observed}}$	${\hat{p}}_{\mathrm{forecasted}}$	$|{\hat{p}}_{\mathrm{observed}}-{\hat{p}}_{\mathrm{forecasted}}|$
${\hat{p}}_{1}$	0.479	0.452	0.027
${\hat{p}}_{2}$	0.495	0.433	0.062
${\hat{p}}_{3}$	0.490	0.500	0.010
${\hat{p}}_{4}$	0.474	0.471	0.003
${\hat{p}}_{5}$	0.423	0.433	0.010
${\hat{p}}_{6}$	0.459	0.365	0.094
${\hat{p}}_{7}$	0.484	0.509	0.025
${\hat{p}}_{8}$	0.469	0.452	0.017
${\hat{p}}_{9}$	0.469	0.404	0.065
${\hat{p}}_{10}$	0.464	0.452	0.012
${\hat{p}}_{11}$	0.500	0.385	0.115
${\hat{p}}_{12}$	0.536	0.942	0.406
${\hat{p}}_{13}$	0.474	0.481	0.005
${\hat{p}}_{14}$	0.459	0.461	0.002

The Euclidean distance of 0.44 was computed using the formula $\sqrt{{\sum \left({\hat{p}}_{\mathrm{observed}}-\;{\hat{p}}_{\mathrm{forecasted}}\right)}^{2}}$.

This small value of Euclidean distance revealed similarity based on spatial distance between observed and forecasted probabilities. A two‐tailed non‐parametric Wilcoxon rank sum test ([38]) with continuity correction was also conducted, at the 5% level of significance, to justify that there does not exist any statistically significant difference between ${\hat{p}}_{\mathrm{observed}}$ and ${\hat{p}}_{forecasted}$. The nonsignificant *p*‐value (*p* = 0.11) with a Wilcoxon test statistic W = 133.5 implies that there exists no significant difference between the true medians of ${\hat{p}}_{\mathrm{observed}}$ and ${\hat{p}}_{\mathrm{forecasted}}.$

Table 10 shows the correlation coefficients between the observed and the forecasted mortalities generated by the MTM. As the table illustrates, all the observed and forecasted mortalities are highly correlated for all CODs, attesting to the validity of the MTM.

**Table 10:** Correlation between observed and forecasted mortality counts for 14 CODs.

COD	ALLCAUSE	NATCAUSE	SEPTICEMIA	MALNEO	DIAMEL	ALZ	INFPNE	CLDR	ODRS	NNSN	SSACL	HD	CD	COVIDUC
**Correlation**	0.78	0.78	0.83	0.64	0.82	0.83	0.83	0.91	0.86	0.89	0.94	0.87	0.87	0.66

## 4 Discussions

The study consisted of three phases. In phase I, a multivariate time series forecasting model was built by taking fourteen CODs. In phase II, the observed and predicted mortality data were combined using Equation 1 to construct the COD Data Matrix (Table 1). In phase III, the COD data matrix was utilized by introducing two new concepts: *Weekly Exceedance in Mortality Count (WEMC)*, and *Weekly Change of Mortality Indicator (WCMI)* to compute different types of simple, and compound probability events related to a single or any combination of the fourteen CODs. If the mortality count at the current week *t* exceeds the mortality count at the previous week (*t* − 1) (described as exceedance in Mortality Count *(WEMC)*), that raises concern from a public health perspective, and it is crucial to investigate how many times such events are occurring. The Weekly Change of Mortality Indicator *(WCMI)* is defined to address such events. The *WCMI* acts as an independent and non‐ identical binomial random variable (RV) for each of the CODs with different probability estimates. It is imperative to compute the probabilities of different possible values that the RV *(WCMI)* ($\alpha_{i}$) might take. Some of these probabilities are computed in Tables 3 and 4. Once the distribution of individual *(WCMI)*s is known, it is possible to compute specific probabilities associated with the sums of multiple independent non‐identical (*WCMI*)s as illustrated in Section 2.1.

As Figure 1 illustrates, many CODs are significantly correlated for the mortality data, and it is crucial to understand the probabilistic structure by computing the joint probabilistic occurrences of the *WCMI*s corresponding to the CODs. For example, from Figure 1 we see that COD due to heart disease ($C_{6}$), and COD due to cerebrovascular Diseases ($C_{7}$) are highly correlated (correlation co‐ efficient = 0.91). From Table 4 we see that the probability of the joint occurrences of both the *WCMI*s exceeding 130 weeks due to the combined effect of heart disease ($C_{6}$), and cerebrovascular Diseases ($C_{7}$) is 37.24%. Similarly, the correlation between the COD due to influenza & pneumonia ($C_{4}$) and the COD due to CLDR ($C_{5}$) was found to be 0.92. From Table 4 we see that the joint probability of *WCMI* greater or equal to 140 due to influenza & pneumonia ($C_{4}$), due to CLDR ($C_{5}$), and due to COVID ($C_{8}$) is approximately 2.63%. Analogous to the joint probabilities, single/individual probabilities can be computed for any of the *WCMI*s corresponding to the CODs. For example, from Table 3 we see that the chance of the event “number of instances where *WEMC* or Ψ occurs due to diabetes mellitus is 141” is 4.63% whereas the chance that the “number of instances where *WEMC* or Ψ occurs due to diabetes mellitus greater or equal to 141” is 52.62%. From the correlation matrix, we also see that mortality due to COVID−19 is strongly and moderately associated with some of the CODs that are evident from other studies [11, 39, 40, 41]. From Table 3 we notice the probability that the “number of instances where *WEMC* or Ψ occurs due to COVID−19 ($C_{8}$) is less or equal to 150” is approximately 0.91, and the chance that it will remain between 140 and 160 is approximately 47%. These probabilities derived from the analytical approach can be crucial for public health researchers and policymakers for informed decision‐making and risk assessment, by controlling the underlying attributes that might be responsible for the probabilities to surge and plummet. The following schematic diagram (Figure 2) illustrates the analytical approach step by step.

The metrics *WEMC* and *WCMI* have important policy implications in the context of public health, particularly in surveillance, resource allocation, and disaster mitigation. The *WEMC* quantifies the weekly mortality exceedance specific to any COD, and the *WCMI* tells us how often such incident happens, offering crucial insights for health policy and public health decision‐making. These tools can be utilized as early warning systems to analyze new health emergencies, such as environmental hazards and infectious disease epidemics, triggering timely research and action. By identifying any spikes in mortality that could be contributing to a burden on the medical system, these measures can also assist policymakers in allocating healthcare resources by enabling them to modify supply chains, hospital capacity, and workforce distribution. Furthermore, by demonstrating the spatial and demographic differences in mortality, these tools might be helpful in offering epidemiological insights and aiding direct focused efforts for communities that are at risk [42].

When it comes to disaster preparedness, *WEMC* and *WCMI* might assist health authorities with enhancing emergency response plans for extreme events, such as pandemics, natural calamities, and catastrophic weather conditions. To provide evidence‐based decision‐making, the computed probabilities, with the help of these analytical tools, also contribute to the evaluation of policy accomplishment by comparing the mortality trends before and after specific interventions such as vaccination campaigns or lockdowns [43].

Health policymakers, and health science researchers might be interested in knowing the probability that the sum of different *α*_*i*_s will take a specific value. For example, in the first row of Table 5, the quantity $P(T_{5}=t)$ computes the probability of the number of instances where *WEMC* or Ψ occurs is 100 due to CODs 1 ‐ 5 ($C_{1},C_{2},\text{.}\text{.}\text{.},C_{5}$). This computation enables us to estimate the probability associated with not only an individual COD but also combining multiple CODs. If time‐dependent mortality data with associated COD is available, the probability of combined/added contribution to any two, or more *(WCMI)*s can be computed. The uncertainty quantification of the CODs can be extended to any quarterly, monthly, and yearly mortality data, and the probability computations can be done in a similar manner as described in the manuscript for weekly data.

Despite being a valuable data set, the CDC provisional count data is subject to some biases, and hence the study findings have a few limitations. First, while reporting the mortalities, there might be reporting delays or time lag bias (geographic variations in real‐time data and backlogs in processing death certificates can occur owing to the delayed reporting methods by some jurisdictions/counties) which might undercount the mortalities in recent weeks. Also, there may be cause‐of‐death misspecification bias. Chronic conditions like COVID‐19, influenza & pneumonia, lower respiratory diseases, cerebrovascular diseases, and heart diseases may be misclassified due to overlapping symptoms. Finally, this data set does not have specific predictors like gender, race, medical history, general health status, insurance status, etc. associated with the CODs to perform predictive modeling to understand how the associated predictors affect each CODs. Utilizing the results obtained from the analytical method, future research should focus more on performing geospatial predictive modeling considering the census tract to explore the impact of social determinants of health and associated risk factors on excess mortality. Exceedance probabilities serve as an analytical approach to evaluate and improve existing mortality modeling forecasting by enabling researchers to explore the extent of variability in exceedance probabilities for different geographic and population sub‐groups, uncovering geographic and socioeconomic inequities. Future epidemiologic research should concentrate more on modern techniques, such as, Bayesian hierarchical models, deep learning models, and ensemble learning techniques ([23, 44]) to account for the complexity in mortality patterns and predict exceedance probabilities in a reliable way, while also utilizing spatial and temporal dependencies.

The computation of probabilities using CDC provisional mortality by selected causes offers a modern analytical approach to understanding mortality rates and their underlying causes. By utilizing this approach, researchers and policymakers can gain valuable insights into trends and patterns in mortality, which can inform public health interventions and resource allocation Liu et al. [45]. Furthermore, the application of this approach to US mortality rates helps to provide a comprehensive understanding of the risks and burdens associated with different COD in understanding and predicting mortality patterns based on specific causes. By delving deeper into the data provided by the CDC, researchers and policymakers can achieve a keen perception into the likelihood of deaths occurring from various causes within a given population. This modern analytical approach allows for the identification of trends and patterns that can inform public health strategies and interventions.

## 5 Conclusion

This study investigates how useful probabilistic inferences can be made corresponding to the different leading CODs by implementing the new analytical methodology. Integrating the concepts of *WEMC*, and *WCMI* with the classic epidemiological set up can not only gain valuable insights into the trends, and patterns of the mortality, but also can play a crucial role in understanding the “exceedance phenomena” with certain degree of accuracy as a function of time.

## Author Contributions

**Aditya Chakrabarty:** conceptualization, methodology, software, data curation, supervision, writing – review and editing, writing – original draft, visualization, validation, formal analysis, investigation. **Mohan D. Pant:** writing, review and editing.

## Conflicts of Interest

The authors declare no conflicts of interest.

## Transparency Statement

The lead author Aditya Chakrabarty affirms that this manuscript is an honest, accurate, and transparent account of the study being reported; that no important aspects of the study have been omitted; and that any discrepancies from the study as planned (and, if relevant, registered) have been explained.

## Data Availability

The data that support the findings of this study are available from the corresponding author upon reasonable request.
